# Supplementary material for: Trust in healthcare providers, information sources, and concerns for new maternal vaccines among pregnant and lactating women in Kenya
Source: PLOS Glob Public Health. 2025 Nov 12;5(11):e0004499. doi: 10.1371/journal.pgph.0004499 (PMC12611154; doi:10.1371/journal.pgph.0004499)
Supplement: Attachment — Submitted filename: PLOS GH Response to Reviewer Comments.docx [file pgph.0004499.s003.docx]

Major Points

Introduction:
• It is imperative for the authors to cite and discuss more than one prior research study addressing the same research problem. This context will underscore the novelty of your study and help differentiate it from previous attempts.

Thank you for your suggestion. We have added all available studies to our knowledge on RSV burden and seasonality in Kenya, as well as studies focused on knowledge and attitudes toward RSV prevention products.

• Clearly state the aim and objectives of your study at the conclusion of the introduction section to provide a concise overview of your research goals.
The last paragraph of the introduction contains the aim of the analysis and was expanded on to provide more clarity on the goals of the manuscript.

Methods:

• The sampling strategy should be clearly defined and reported in the methods section.

Thank you for noting this oversight, we have added information on the sampling strategy to the methods section as advised.

• Authors should clarify how the sample size was determined.

Thank you for this comment. We have added detail on the inputs used to calculate sample size for this survey.

• Line 110 “Data collectors completed a standard training, and the survey instrument was pretested.” What does ‘standard’ mean? What did they do in the training and what made you sure they were ready to collect data?

Thank you for this comment. We have added information related to what was included in the training.

• The authors fail to provide any information in their manuscript that would allow readers to understand how the sample differs from the target population, particularly in terms of key sociodemographic characteristics such as age, gender, and others. This lack of information raises concerns about the representativeness of the sample.

The target population is pregnant and lactating women in Kenya. We aimed to include women from both urban and rural settings, assuming that many of the factors of interest may differ between these two subgroups. This has been added to the methods for clarity. Given the high level of facility deliveries in these regions, we feel this is an ideal recruitment strategy for the purposes of this analysis.

The sample is described in Table 1 and includes the sociodemographic variables that were collected during the study. The limitations section of the discussion addresses bias that may have arisen by recruiting from health facilities only as well as our attempts to obtain a representative sample. Recruitment methods were constrained by study budget, timelines, and ethical considerations.

• Authors should clarify how they estimated the reliability or internal consistency of the questionnaire used, using, for example, Cronbach’s alpha to measure whether a score is reliable.

Thank you for this comment. We have added language to explain how our questionnaire was developed and validated, including constructs grounded in establish vaccine hesitancy frameworks and theories. These survey tools are commonly utilized and adapted for studies of vaccine hesitancy. Because we drew on validated tools for this study, we did not assess reliability nor internal consistency for this questionnaire. However, we have noted this as an important limitation of the study and recommend further study to refine the scale for assessing maternal vaccine knowledge, attitudes, and behaviors.

• More information should be provided regarding the considerations concerning the literature review (please add the references) and the authors' previous research experience in developing the questionnaire.

The literature review in the introduction has been updated. We have also included some background on the theories and items that were used in developing the questionnaire.

• The study should use additional statistical analysis. Specifically, information on how the authors assessed the normality of numeric variables and any relevant assumptions made for the chosen statistical tests or models should be reported to enhance the transparency and reliability of the statistical analysis. Also, information should be added regarding the logistic regression analysis used (e.g., independent and dependent variables, assumptions, etc.)

Thank you for this, we agree that more details of the analysis done are needed. These have been added to the methods section. Of note, there were no numeric variables collected or used in this study and the study aims are descriptive in nature as amended in the introduction of the manuscript. Therefore, we feel the existing analysis is appropriate for study aims and including a regression analysis would not be in line with the scope of the descriptive aims.

• Line 139-140 “New vaccine concerns. We asked question about future vaccine concerns and asked 140 participants to rank their answers from 1-6,…”. Not clear whether the ranking is a score or the number of options, if number of options 7 were actually given.

This has been clarified, the number of options was incorrectly listed as 6 and has been resolved in the manuscript and the ranking of the question responses was clarified.

Results:
• Detailed findings from the statistical analyses used such as the logistic regression models should be reported in the text of the Results section. The authors should create new tables reporting the results from these analyses, which should be included among the main tables of the manuscript.

No regression models were done in this study, the purpose of the manuscript is descriptive in nature. This has been clarified in the introduction.

Discussion:
• The discussion section does well to explore the underlying meaning of the research and its potential implications in other areas of study. The authors have discussed how their findings contribute to the existing knowledge and highlight the significance of their research within a broader context. However, they should consider addressing possible improvements or future directions that can further develop the concerns of their research.

Thank you – we have included some ideas for future directions through the discussion.

• Lines 195-197 “While these specific responses were about new maternal vaccines broadly, the questions were prompted by describing maternal RSV vaccines.” Critically state if you feel this made any difference.

Thanks for flagging this – we have added how this framing could have influenced how participants answered.

• Lines 199-208 “While research on maternal RSV vaccines in low- and middle-income countries is lacking, there have been a few studies focused on knowledge and acceptance of RSV vaccines in pregnancy. A study among Australian women focused on RSV and Group B Streptococcus (GBS) vaccines in pregnancy found that safety data was the most important factor for acceptance (17). These findings align with ours as side effects, an important measure for safety, was the biggest concern among women in both counties. A sample of women in Jordan also found favorable opinions about RSV vaccination during pregnancy, given the vaccine is safe (18). The concern of vaccine ingredients, which emerged as the second most important in both counties, was not as prevalent in other studies.”. You begin the paragraph talking about low- and middle-income countries but go on to discuss Australia and Jordan instead.

Thank you for flagging this – we have included information as to why we included these examples – is it due to the fact that there are no studies related to this from low- and middle-income countries.

• Lines 240-251 “RSV vaccine is recommended in the third trimester, a relatively narrow window 241 compared to other vaccines in pregnancy (i.e., tetanus toxoid) which may be given at 242 any time in pregnancy. A study in Kenya found that two in three pregnant women had 243 their first antenatal care (ANC) visit prior to this window (29). This may lay the 12 244 groundwork for healthcare providers to prepare pregnant women for newer maternal 245 vaccines by providing information and addressing concerns during their first ANC visits, 246 before they are eligible to receive these vaccines. Our study found that media is a 247 trusted information source among pregnant and lactating women. Targeted media and 248 social media campaigns in advance of RSV vaccine availability should include 249 messages emphasizing the opportunity for mothers to protect their newborns by being 250 vaccinated themselves, and including information about vaccine safety as well as 251 efficacy (30).” You begin the paragraph talking about uptake of RSV vaccine and then sharply switch to discussion about the media. These should be two separate paragraphs expanded on and discussing other published research.

Thank you for this comment. We have separated these two discussion points into separate paragraphs, per your recommendation, and have added additional commentary on related literature for each.

Reviewer #2: This cross-sectional survey aimed to understand decision-making factors among pregnant and lactating women for receiving a newly licensed vaccine during pregnancy. As far as respiratory syncytial virus (RSV) is a leading cause of acute lower respiratory infections among infants under six months of age, it is important to conduct cross-sectional survey to understand which information sources pregnant women trusted and their intentions toward future maternal vaccines given in pregnancy, and conducting survey will have an integral role in the working process of determining appropriate measures required to equip healthcare providers with salient and digestible information to be able to strongly recommend a future maternal RSV vaccine, and pregnant and lactating women rely on trusted sources to inform their decision-making related to maternal vaccines.

The manuscript is technically sound, and the data supports the conclusions, and it describes methodologically and ethically rigorous research with conclusions that are appropriately drawn based on the data presented. Furthermore, conclusions are presented in an appropriate fashion and are supported by the data, and the data presented in the manuscript supported the conclusions drawn, and the authors did not overstate their conclusions. The language in the submitted article is clear, correct, and unambiguous and is written in standard English. The research meets all applicable standards for the ethics of experimentation and research integrity. The article adheres to appropriate reporting guidelines and community standards for data availability. Moreover, the manuscript is well organized and written clearly enough to be accessible to non-specialists.

Generally, the article is suitable for publication subject to the following minor corrections or comments for revision and submission to current or other journals. Here below I tried to put my concerns!

Abstract:
Dear author, in the abstract section, you stated that pregnant and lactating women from two counties in Kenya, Nakuru and Mombasa, were recruited to complete a cross-sectional survey in August-September 2022. However, in the methods section, you stated that data collection occurred from 25/07/2022 through 21/09/2022. It is recommended that the author should report consistently.

Thank you for your comment and catching this detail. The dates presented in the methods section are correct and we have edited this to match in the abstract.

Another concern in the abstract section: it is better to include an adjusted odds ratio (AOR) with a 95% confidence interval (CI) which was used to measure the strength of the association between factors and the outcome variable. In addition, it is better to include the statistical software used for data entry and analysis and the sampling methods used to recruit pregnant and lactating women from two counties in Kenya, Nakuru and Mombasa, to complete a cross-sectional survey.

Thank you for this comment, however, a regression model was not included in this study given the study aims.

Methods:
Dear author, You stated that participants were recruited from 20 health facilities in Nakuru County (rural) and Mombasa County (urban). A range of health facility types was included, from level 2 through level 5 (line numbers 107-109). However, you did not provide in detail the sampling technique and procedure employed to arrive at the desired sample size (400 pregnant and lactating women).

Thank you for noting this oversight, we have added information on the sample size calculation and sampling strategy to the methods section as advised.

Furthermore, it is recommended to provide the sample size determination, data processing and analysis used in this study.

Thank you for noting this, we have added the sample size determination to the methods.

Results:
Dear author, You reported that nearly half of participants had at least a secondary education (line number 150). However, Table 1 showed that the percentages of participants who completed a secondary education in Nakuru and Mombasa counties were 33% and 27.5%, respectively. It is better to revise it.

Thank you for pointing this out. The text was clarified to show that the “nearly half” in the text includes those with a secondary education AND those with college level education, in which case it is 58.5 % in and Nakuru and 54% in Mombasa, which are both more than half.

Regarding trust in information from traditional media, 31% disagreed and 68% agreed for Nakuru participants (Figure 1). The summation of participants who disagreed and agreed was 99%. Dear author, the summation of participants should be 100%. It is recommended that the author should revise Figure 1.

This is a rounding error. When all values include a 0.5 decimal, and a standard of rounding is used (such as 0.5 is always rounded up or always rounded down), these rounding errors are inevitable. We feel strongly that a rounding standard is needed and added a footnote to the figure to explain the rounding error.
